# Supplementary material for: Metal-Induced Energy Transfer (MIET) for Live-Cell Imaging with Fluorescent Proteins
Source: ACS Nano. 2023 Mar 30;17(9):8242–51. doi: 10.1021/acsnano.2c12372 (PMC10173696; doi:10.1021/acsnano.2c12372)
Supplement: Supplementary file 1 — nn2c12372_si_001.pdf [file nn2c12372_si_001.pdf]

# Supporting Information: Metal-Induced Energy Transfer (MIET) for Live-Cell Imaging with Fluorescent Proteins

Lara Hauke,[†,¶] Sebastian Isbaner,[†,¶] Arindam Ghosh,[†,¶] Isabella Guido,[‡] Laura Turco,[‡] Alexey I. Chizhik,[†] Ingo Gregor,[†] Narain Karedla,[*,†] Florian Rehfeldt,[*,†] and Jörg Enderlein[*,†,§]

†*Third Institute of Physics – Biophysics, Georg August University, Friedrich-Hund-Platz 1, 37077 Göttingen, Germany*

‡*Max Planck Institute for Dynamics and Self-Organization, Am Faßberg 17, 37077 Göttingen, Germany*

¶*Contributed equally to this work*

§*Cluster of Excellence "Multiscale Bioimaging: from Molecular Machines to Networks of Excitable Cells" (MBExC), Universitätsmedizin Göttingen, Robert-Koch-Str. 40, 37075 Göttingen, Germany*

E-mail: narain.karedla@kennedy.ox.ac.uk; florian.rehfeldt@uni-bayreuth.de; jender@gwdg.de

# S.1. Determining a Molecule's Height Using the Average Fluorescence Decay-Time and MIET

To evaluate, whether it is justified to use the average fluorescence decay-time for a simplified analysis of bi-exponential fluorescence decays we consider two scenarios. In the first case, we assume that the two decay rates are caused by different non-radiative rates $k_{\mathrm{nr},1}$ and $k_{\mathrm{nr},2}$ while the fluorescence rate $k_{\mathrm{fl}}$ is the same for the two populations. In the second scenario, we assume that the quantum yield of both populations are the same. These two scenarios are the limiting cases for the reduction of the decay time. The first is accounting any reduction to an increased non-radiative rate $k_{\mathrm{nr}}$, whereas the latter attributes this effect to an increase of the radiative rate $k_{\mathrm{fl}}$. Also, in what follows we always assume that the emission spectrum is the same for both populations. The assumed numbers for the simulated results correspond to the range found in the experiments shown in this manuscript. However, the considered distributions are generally wider than found in the real experiments.

The results show, that we can safely use the selected FP for MIET. However, this will not hold for any FP in general. Since the error in height estimation depends in a peculiar way on the quantum yield and the respective contribution of the two states, the error can become more significant. As a rule of thumb, a high total quantum yield is preferred and the contribution of the second decay-time should be as small as possible. It is advisable to follow a similar analysis as shown here to estimate the errors of the MIET calibration.

## S.1.1. Constant Fluorescence Rate

From these assumptions, it follows that the decay times for the two are

$$\tau_1 = (k_{\mathrm{fl}} + k_{\mathrm{nr},1})^{-1} \text{ , and } \tau_2 = (k_{\mathrm{fl}} + k_{\mathrm{nr},2})^{-1} \text{ .}$$

The quantum yields are $\eta_1 = k_{fl}\,\tau_1$ and $\eta_2 = k_{fl}\,\tau_2$, respectively. If we have mixture with the relative amounts $N_1$ and $N_2$ of both species, we will observe a fluorescence decay as

$$I(t) = A_1 \exp\left(-\frac{t}{\tau_1}\right) + A_2 \exp\left(-\frac{t}{\tau_2}\right) \ ,$$

where $A_1 = N_1\eta_1$ and $A_2 = N_2\eta_2$. The average decay-time and quantum yield of this mixture will be

$$\overline{\tau} = A_1\tau_1 + A_2\tau_2 \ \text{, and}$$

$$\overline{\eta} = A_1\eta_1 + A_2\eta_2 \ .$$

These values allow us to calculate the 'MIET curves' for population 1, population 2, and the average of the two. Figure S1 shows the three curves for parameters that exceed the values that we observe in the case of lifeact-mScarlet or GFP (see sections S.2 and S.3, respectively).

These curves show how the decay-times drop near the surface, but it is important to note that this is due to an increase of the radiative rate. Therefore, we need to determine the rate $k_{fl}(z)$ and accordingly the resulting quantum yield $\eta_{1,2}(z)$ as a function of the height. These dependencies are shown in Figure S2.

Using the $z$ dependent values of the quantum yields, we can now determine the average decay-time $\overline{\tau}(z)$ that a population of $N_1$ and $N_2$ molecules at the height $z$ will show. Finally, we use $\overline{\tau}(z)$ to compute the height value $\overline{z}$ based on the MIET curve that was calculated from the average free space lifetime (green curve in Figure S1). The difference of $\overline{z}$ to the true value $z$ will show the expected error. As shown in the top panel of Figure S5 the maximal error is about $\Delta z = 2$ nm.

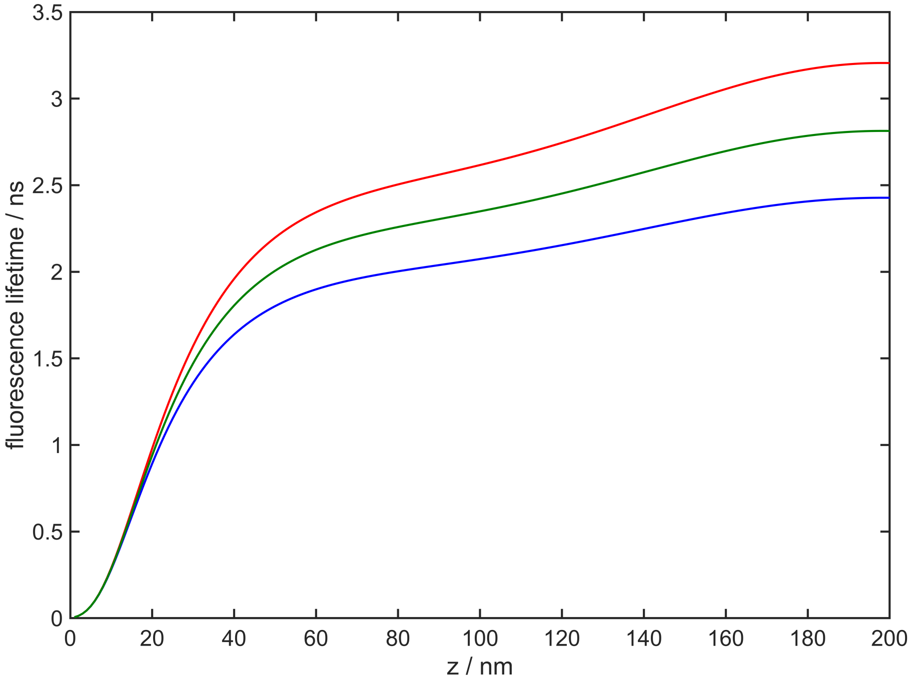


Figure S 1: Fluorescence decay-time as a function of the height over a MIET surface for different quantum yields. Red: $\tau_1 = 3.00$ ns, $\eta_1 = 0.70$, blue: $\tau_2 = 2.30$ ns, $\eta_2 = 0.54$, and green: $\overline{\tau} = 2.65$ ns, $\overline{\eta} = 0.62$. The green curve was obtained by a mixture of $N_1 = 43.5$ % and $N_2 = 56.5$ %.

## S.1.2. Constant Quantum Yield

Again, we are starting our analysis considering the decay-times of the two populations

$$\tau_1 = (k_{\mathrm{fl},1} + k_{\mathrm{nr},1})^{-1} \quad \text{, and } \tau_2 = (k_{\mathrm{fl},2} + k_{\mathrm{nr},2})^{-1} \quad .$$

The quantum yield is $\eta = k_{\mathrm{fl},1}\,\tau_1 = k_{\mathrm{fl},2}\,\tau_2$, respectively. If we have mixture with the relative amounts $N_1$ and $N_2$ of both species, we will observe a fluorescence decay as

$$I(t) = A_1 \exp\left(-\frac{t}{\tau_1}\right) + A_2 \exp\left(-\frac{t}{\tau_2}\right) \quad ,$$

where $A_1 = N_1$ and $A_2 = N_2$, and the average decay-time being $\overline{\tau} = A_1\tau_1 + A_2\tau_2$.

Figure S3 shows the MIET curves of population 1, population 2, and the average of

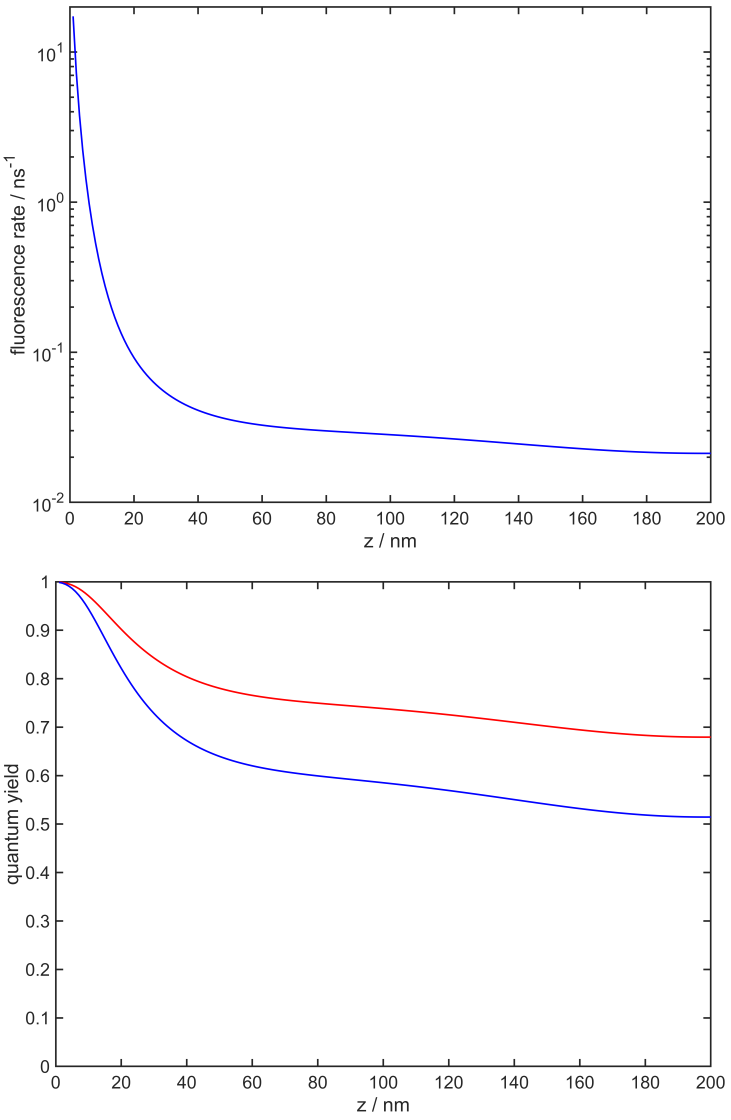


Figure S 2: Emission rate $k_{\mathrm{fl}}(z)$ (left) and fluorescence quantum yield $\eta(z)$ (right) as a function of the height over a MIET surface. Red: $\tau_1 = 3.00$ ns, $\eta_1 = 0.70$, blue: $\tau_2 = 2.30$ ns, $\eta_2 = 0.54$. The given numbers hold for the molecules in homogeneous solution. Because for both molecules, $k_{\mathrm{fl}}$ is the same in the solution, this is also true above the surface.

the two using parameters that exceed the values that we observe in the case of mScarlet or GFP (see sections S.2 and S.3, respectively). Next, we determine the rates $k_{\mathrm{fl},1}(z)$ and $k_{\mathrm{fl},2}(z)$ as well as the resulting quantum yield $\eta_{1,2}(z)$ as a function of the height. These dependencies are shown in Figure S4. Because for both molecules the quantum yield is the same in the solution, this is also true above the surface. Using the $z$ dependent

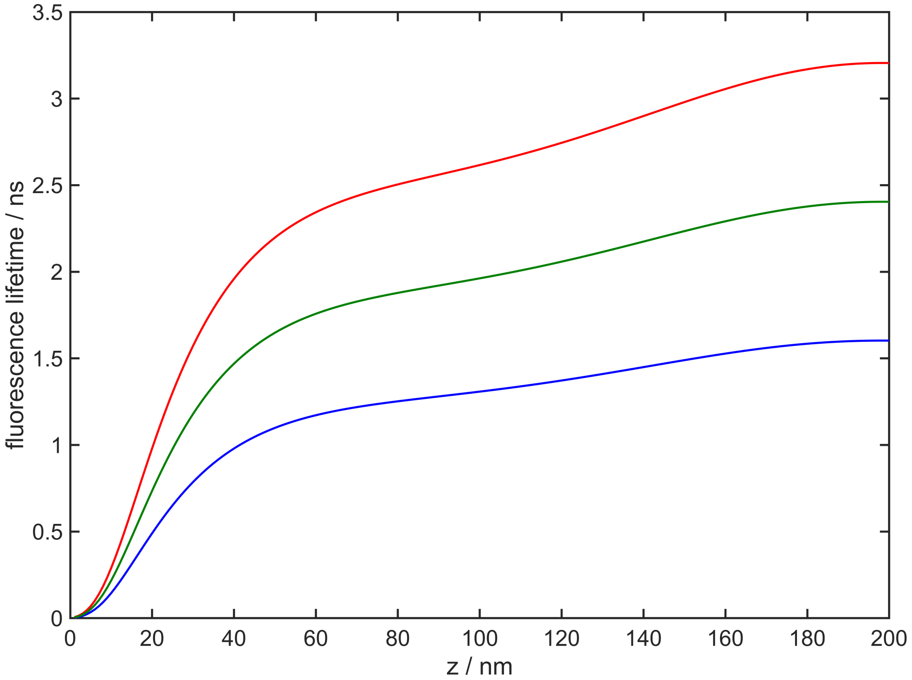


Figure S 3: Fluorescence decay-time as a function of the height over a MIET surface for the following parameters: $\eta = 0.70$, $\tau_1 = 3.00$ ns (red), $\tau_2 = 1.50$ ns (blue), and $\overline{\tau} = 2.25$ ns (green).

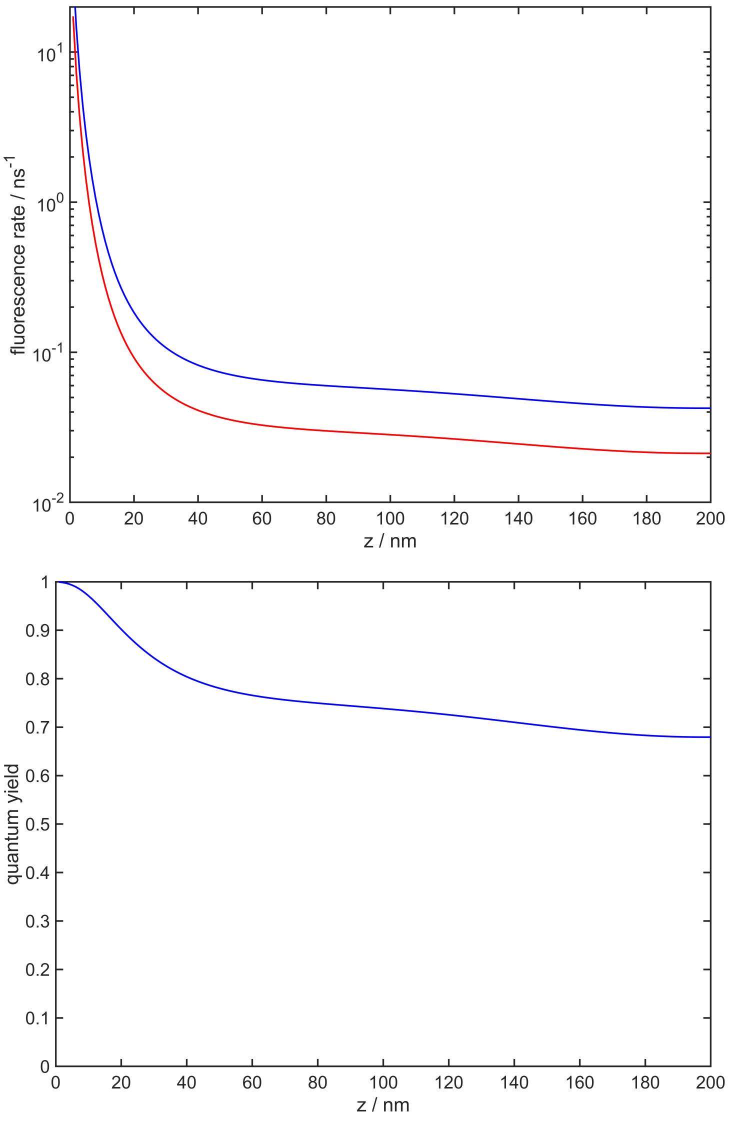


Figure S 4: Emission rate $k_{\text{fl}}(z)$ (top) and fluorescence quantum yield $\eta(z)$ (bottom) as a function of the height over a MIET surface. Free space quantum yield is $\eta = 0.70$. Red: $\tau_1 = 3.00$ ns, blue: $\tau_2 = 1.50$ ns. The given numbers hold for the molecules in homogeneous solution.

values of the quantum yields, we can now determine the average decay-time $\overline{\tau}(z)$ that a population of $N_1$ and $N_2$ molecules at the height $z$ will show. Finally, we use $\overline{\tau}(z)$ to compute the height value $\overline{z}$ based on the MIET curve that was calculated from the average free space lifetime (Figure S3). The difference of $\overline{z}$ to the true value $z$ will show the expected error. As shown in the right panel of Figure S5 there is no error in the

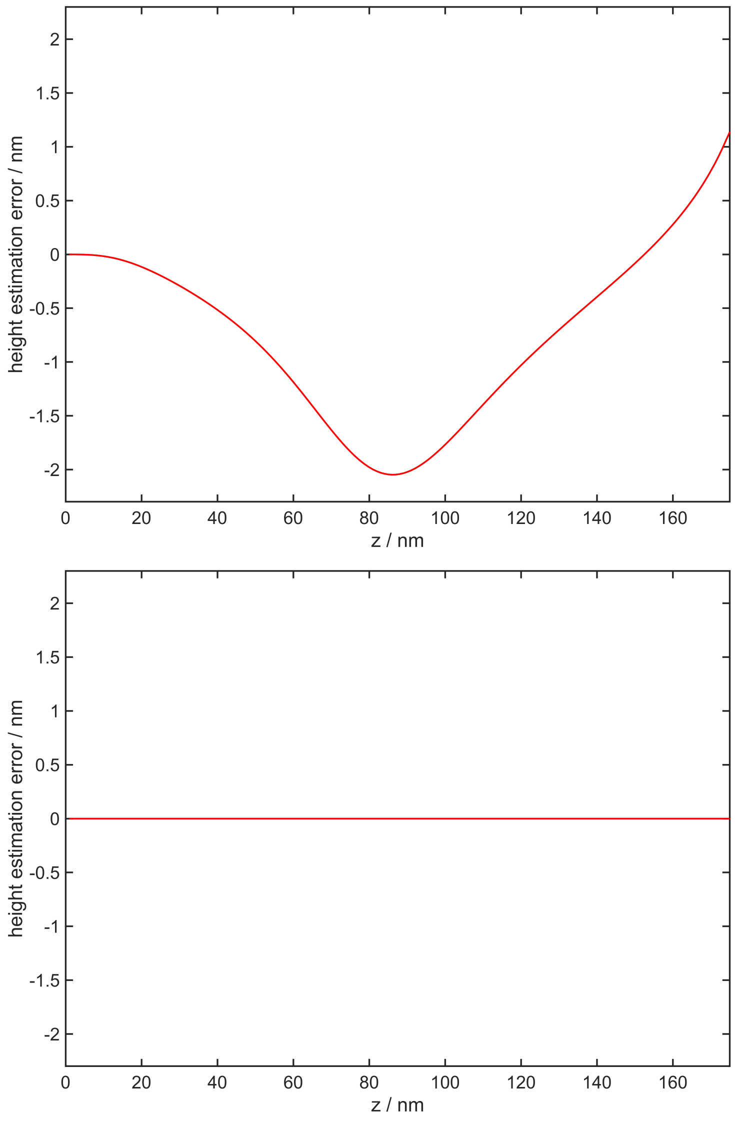


Figure S5: Error of the estimated height ($\Delta z = \overline{z} - z$) due to analysis based on the average fluorescence decay-time. Top: assuming same fluorescence rate; bottom: assuming same fluorescence quantum yield of both populations.

determination of the height. We therefore can safely assume that the average lifetime introduces no significant error in the MIET evaluation.

## S.2. GFP Lifetime And Calibration Curves

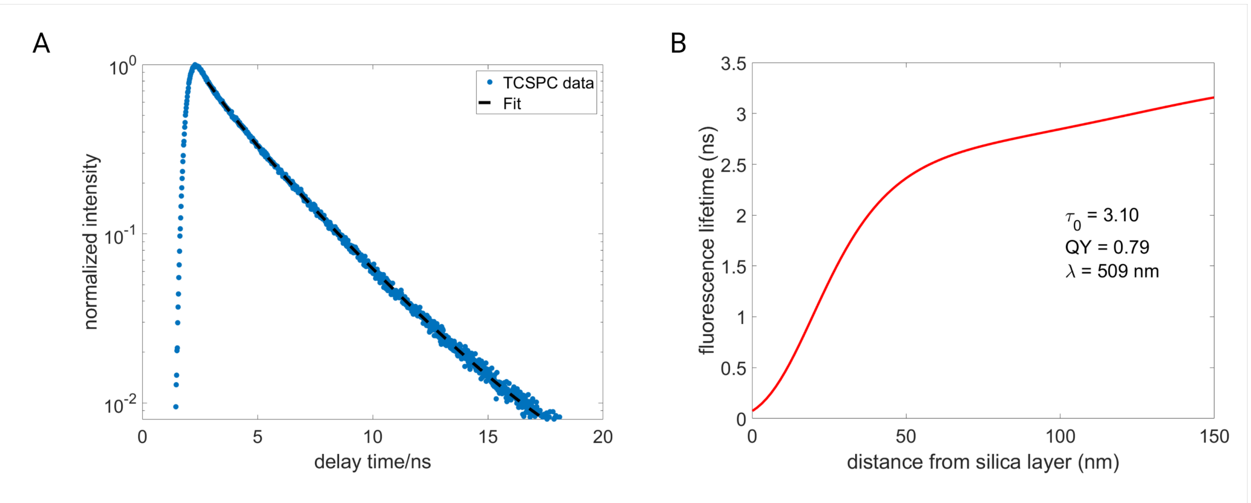


Figure S 6: **A**: Free space lifetime of GFP in *D.d.* cells. TCSPC histogram on glass and tail-fit of the decay is shown. We performed a bi-exponential tail-fit to obtain lifetime values of 3.3 ns and 1.3 ns with fit amplitudes of 0.88 and 0.12 respectively. The average lifetime was calculated to be 3.1 ns which was used to compute the MIET calibration curve shown in **B**. **B**: MIET calibration curve as obtained from experimentally determined free-space lifetime $\tau_0$ = 3.1 ns, quantum yield QY = 0.79 and at emission wavelength $\lambda$ = 509 nm.

The excited-state fluorescence lifetime and fluorescence quantum yield (QY) of GFP are known to be 2.5 ns and 0.79, respectively.[1,2] In this study, we obtain a free-space fluorescence lifetime value $\tau_0$ = 3.1 ns for cAR1-GFP in *D.d.* cells. For the calculation of MIET calibration curve (Figure S6B), although we used the value of $\tau_0$ as 3.1 ns, at the same time we used a fluorescence quantum yield (QY) of 0.79 which was previously reported and not measured independently for this work. With an increase in $\tau_0$ from 2.5 ns (reported value) to 3.1 ns (current study), it is evident that the QY values will also rise following the empirical relationship between fluorescence lifetime and quantum yield (assuming that the radiative rate remains constant).[3] In order to estimate possible changes in axial distances due to an increase in QY values, we computed calibration curves considering theoretical QY values of 0.85, 0.90, 0.95, and 1.0 in addition to the existing QY of 0.80 (see S7A). Next, we estimated the heights of the same developed *D.d.* cell reported

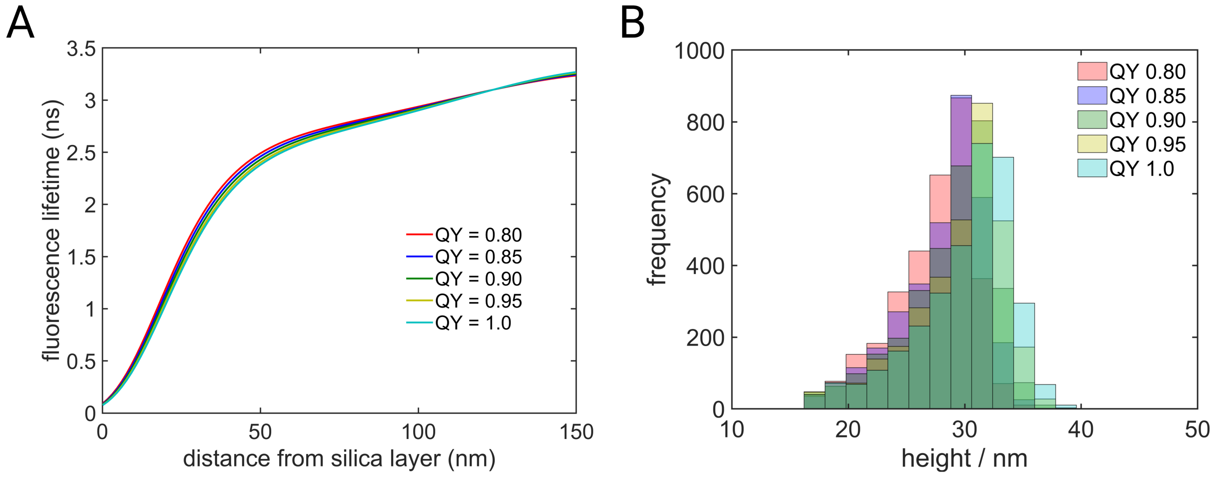


Figure S 7: **A**: MIET calibration curves calculated for five different quantum yield (QY) values of 0.80, 0.85, 0.90, 0.95, and 1.0 for GFP in *D.d.* cells using free-space lifetime $\tau_0$ = 3.1 ns and emission wavelength $\lambda$ = 509 nm. The calibration curve corresponding to QY = 0.80 is the same as shown in Figure 6A. **B**: Corresponding height values for a developed *D.d.* cell same as Figure 2B in the main text (red bar histogram plot, QY = 0.80) and comparative heights for other QY values as calculated from the respective calibration curves in **A**. As demonstrated, across QY values from 0.80 to 1.0, we obtain the following heights: 27 $\pm$ 3 nm (QY 0.80), 28 $\pm$ 3 nm (QY 0.85), 29 $\pm$ 4 nm (QY 0.90), 29 $\pm$ 4 nm (QY 0.95), and 30 $\pm$ 4 nm (QY 1.0).

in Figure 2 of the main text using each of these calibration curves. We obtained axial distances of 27 $\pm$ 3 nm (QY 0.80, already reported in Figure 2B of main text), 28 $\pm$ 3 nm (QY 0.85), 29 $\pm$ 4 nm (QY 0.90), 29 $\pm$ 4 nm (QY 0.95), and 30 $\pm$ 4 nm (QY 1.0). Hence, we observe a maximum shift of 3 nm in mean height value from QY = 0.80 to the maximum possible QY = 1.0. A shift of 3 nm in mean height is well within the experimental error bounds of MIET experiments. Therefore, we can safely use the previously reported experimentally measured QY of 0.79/ 0.80 for GFP in case of *D.d.* cells for this study as well.

### S.2.1. Fluorescence lifetime values of GFP in *D.d* measurements

FLIM images and corresponding lifetime histograms for GFP-labeled *D.d* are presented in Figure S8.

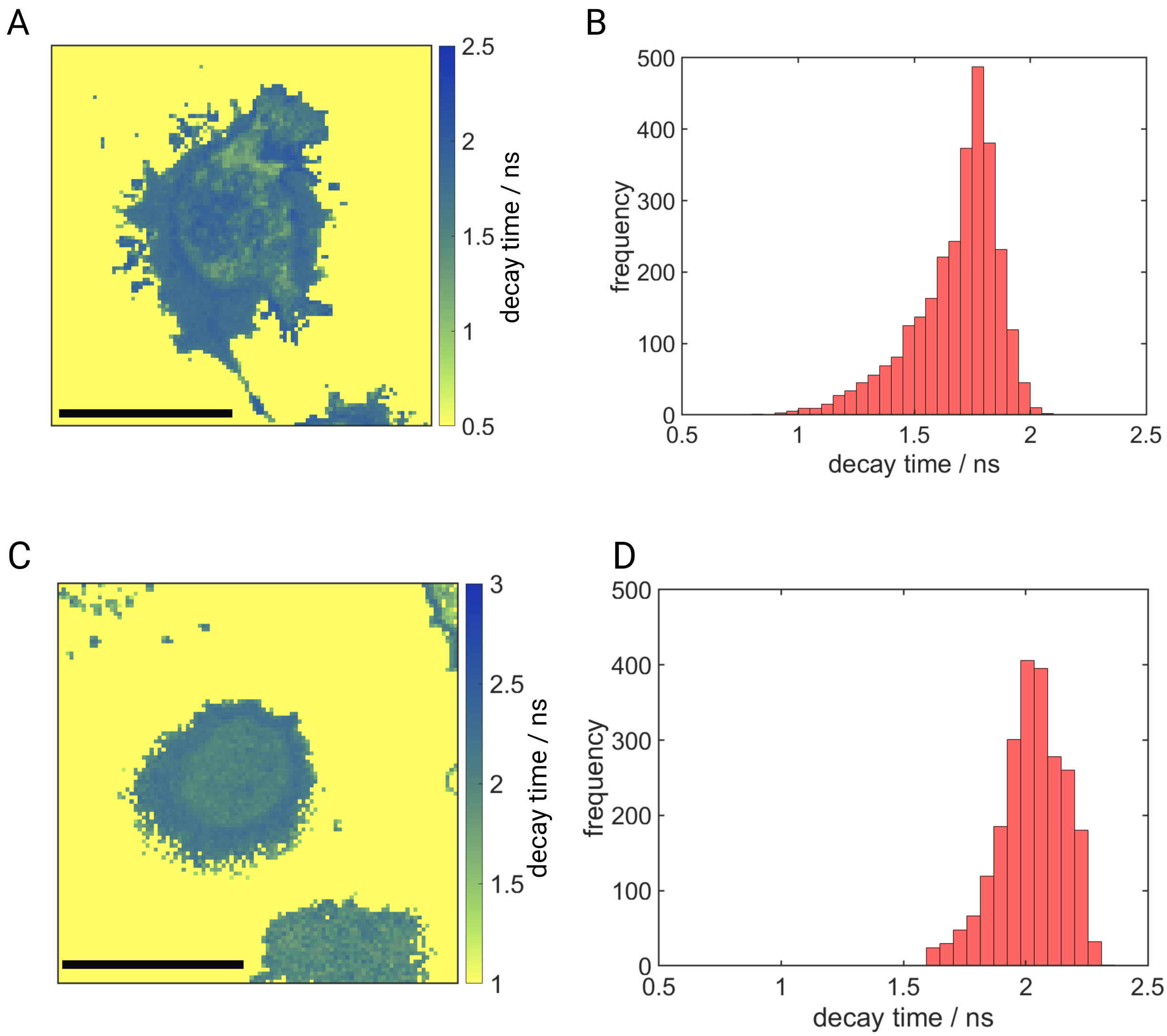


Figure S8: **A**: FLIM image from of a recorded spreading *D.d.* cell in its developed stage pulsed with cAMP. Axial distances of the same cell are presented in Figure 2 in the main text. **B**: Fluorescence lifetime values as obtained from **A**. **C**: FLIM image from a *D.d.* cell in its vegetative stage. Axial distances of the same cell are presented in Figure 2 in the main text. **D**: Fluorescence lifetime values as obtained from **C**. Scale bar 10 μm

## S.3. lifeact-mScarlet lifetime and calibration curves

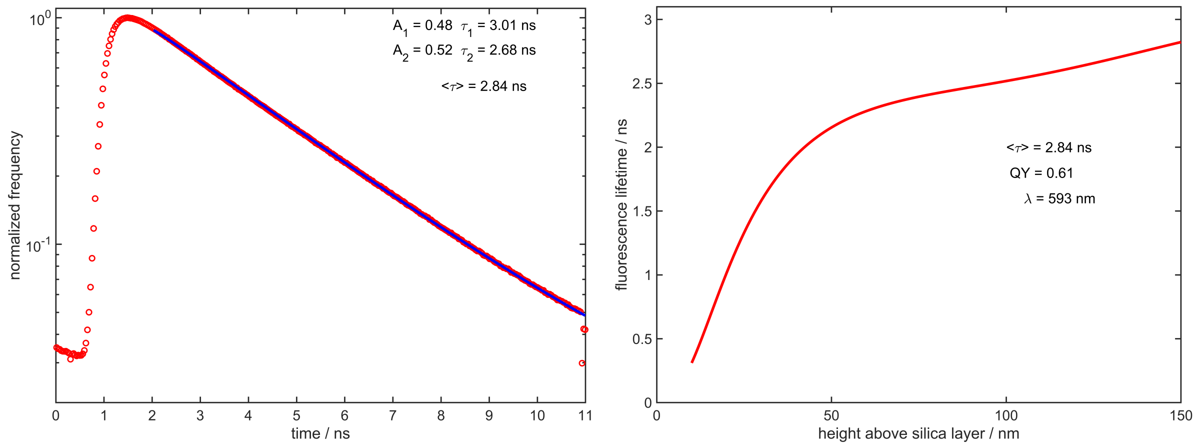


Figure S 9: Left: TCSPC histogram of lifeact-mScarlet in SAOS-2 cells on glass. We performed a bi-exponential tail-fit to obtain lifetime values of 3.0 ns and 2.7 ns with amplitudes of 0.48 and 0.52, respectively. The average lifetime was calculated to be 2.84 ns which was used to compute the MIET calibration curve. Right: MIET calibration curve for lifeact-mScarlet as obtained from experimentally determined free-space lifetime of 2.84 ns, quantum yield QY = 0.61 and at emission wavelength $\lambda$ = 593 nm.

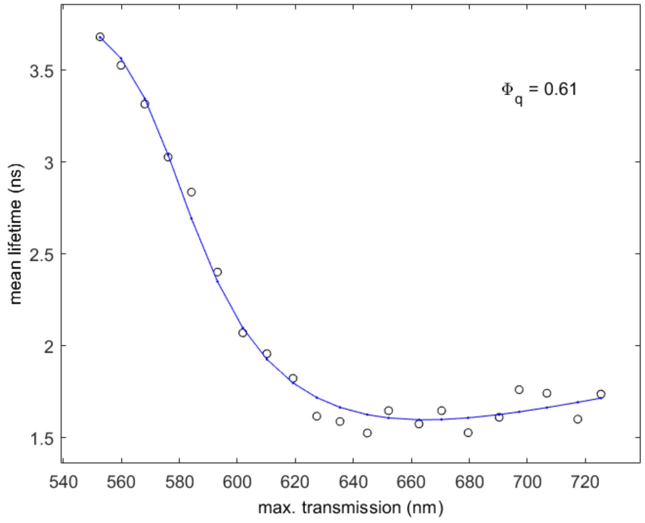


Figure S 10: Fluorescence quantum yield measurement of lifeact-mScarlet. Shown is the spectral dependence of the fluorescence decay-time of the fluorescent protein in a nanocavity. From the specific dependence one can accurately determine the fluorescence quantum yield. For details, see ref.[3]

The excited-state fluorescence lifetime and fluorescence quantum yield (QY) of mScarlet are reported to be 3.9 ns and 0.70, respectively.[4] For this study, we measured these values for our lifeact-mScarlet construct within SAOS-2 cells. We obtain a free-space fluorescence lifetime value of $\langle\tau\rangle = 2.84$ ns and a quantum yield of 0.61. (see Figures S9 and S10). These values were used in combination of the emission spectrum to calculate the MIET calibration curve for lifeact-mScarlet that was used to determine the height values. The quantum yield was determined using the method reported by Ruhlandt.[3]

# S.4. MScarlet Lifetime Comparison

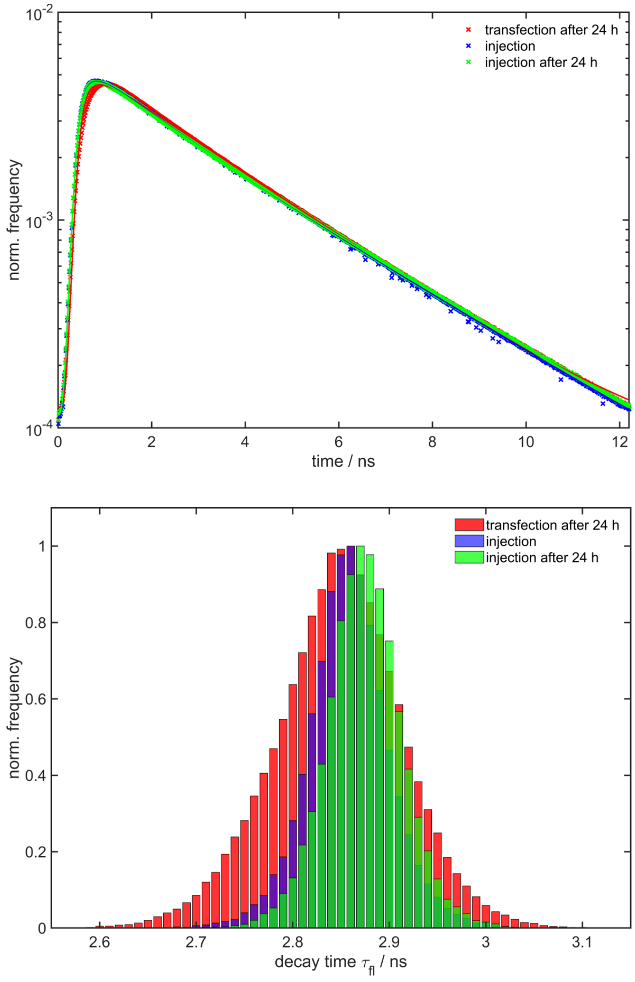


Figure S 11: Top: Fluorescence decays of lifeact-mScarlet in SAOS-2 cells. The fitted average decay-times are 2.68 ns for the transfected cells, 2.81 ns for the cells directly after micro-injection of purified protein, and 2.86 ns at 24 h after micro-injection. Bottom: Histograms of pixel-integrated fluorescence decay-times of the same samples. The histograms show mean decay-times of $2.85 \pm 0.07$ ns, $2.86 \pm 0.04$ ns, and $2.87 \pm 0.04$ ns, respectively. One may note, that the distribution in the case of the transfected cells is broader and shows a tail towards lower decay-times as compared to the micro-injected cells.

For some experiments it may be difficult or non-desirable to transfect cells with a fluorescent protein. In these cases, a viable option might be to micro-inject the protein into the cells. In order to assess this possibility, we compared the fluorescence lifetimes of lifeact-mScarlet in SAOS-2 cells (passage #16, ACC 243, DSMZ). One batch of cells was

transfected as described earlier and FLIM images were recorded 24 h post transfection. A second batch was grown in parallel without transfection. Prior to measurement, we used a Transjector 5246 (Eppendorf, Hamburg, Ger, 5246 01084) and Femtotips (Eppendorf, Hamburg, Ger, 930000035) to inject about 500 pL of a purified solution of lifeact-mScarlet-$His_6$ (0.78 mg/ml in elution buffer (50 mM Tris/Cl pH 8.0, 250 mM NaCl, 10 mM $\beta$-Mercaptoethanol, 250 mM Imidazol) with 2% sucrose) into cells. FLIM images of these cells were recorded in the same way as for the transfected cells. One set of images was recorded directly after micro-injection, a second set was recorded 24 h after micro-injection.

A comparison of the obtained results is shown in Figure S11. The obtained fluorescence decay times agree very well, showing that both methods of live cell staining work. The distribution of the decay-times in the transfected cells is broader than that for the micro-injected cells. Reasons for this might be the more uniform protein distribution in micro-injected cells, or a more homogeneous state of maturation of the purified fluorescent protein. In the transfected cells, there is continuous synthesis (and degradation) of proteins in the cytoplasm. This may increase the inhomogeneity of the fluorescence properties.

# S.5. Additional Data And Videos

## S.5.1. SAOS-2 Video

Video S1 shows part of a migrating SAOS-2 cell 96 hours post transfection with lifeact-mScarlet. Continuous live-cell MIET measurements were done with a 600$\times$600 pixels (corresponding to 120 $\mu$m$\times$120 $\mu$m) field of view and stopped after 7.5 hours.

## S.5.2. *D. discoideum* Videos

Videos S2 and S2e show proliferation and membrane dynamics of developed *D.d.* cells pulsed with cAMP. Movie S2 corresponds to Figure 2 displayed in the main text. Video S2e illustrates the error values in height determination per pixel of movie S2. The false color scale represents height values from the gold surface. Videos S3 and S3e illustrate dynamics of *D.d.* cells in their vegetative stage. Video S3 corresponds to Figure 2 displayed in the main text. Video S3e illustrates the error values in height determination per pixel of movie S2. False color scale represents axial distances from the gold surface. All Videos are played at 30 fps for better visualization.

# S.6. TCSPC Data Of Fluorescent Proteins

Figures S12–15 show TCSPC histograms of fluorescent proteins that were assessed for our studies on live-cell MIET.

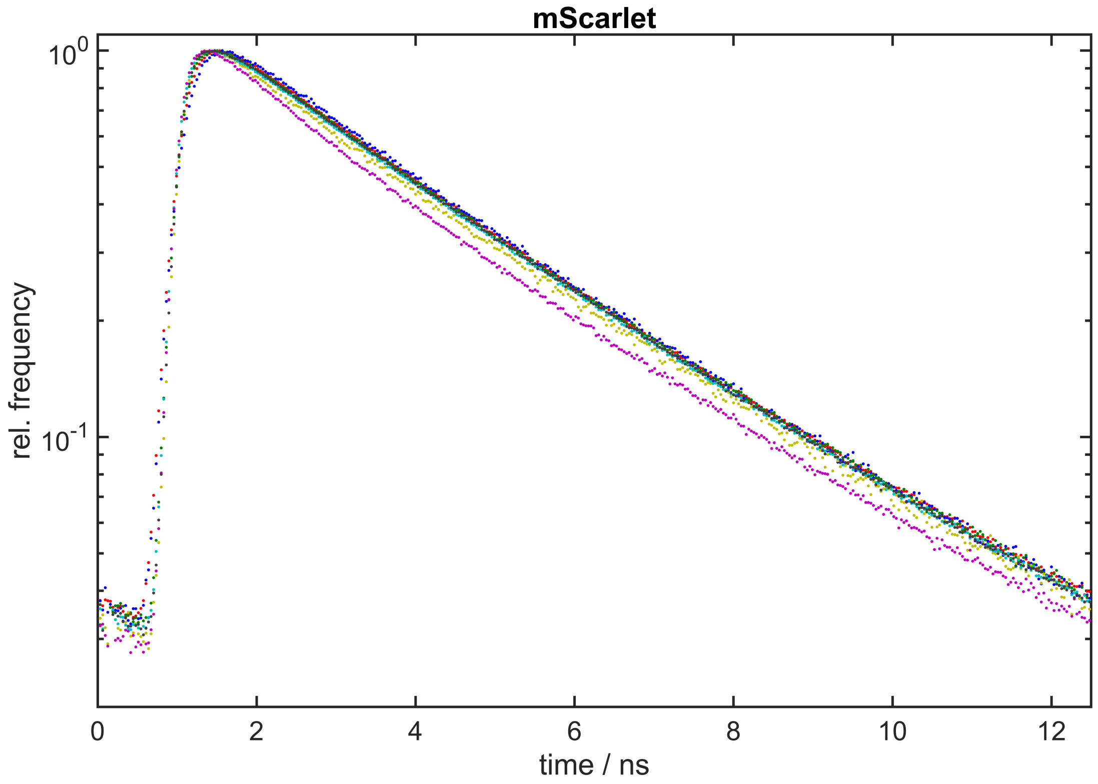


Figure S 12: Fluorescence decays of lifeact-mScarlet in SAOS-2 cells. In the cells lifeact-mScarlet shows a decay with an average decay-time of 2.8 ns that varies only slightly from cell to cell.

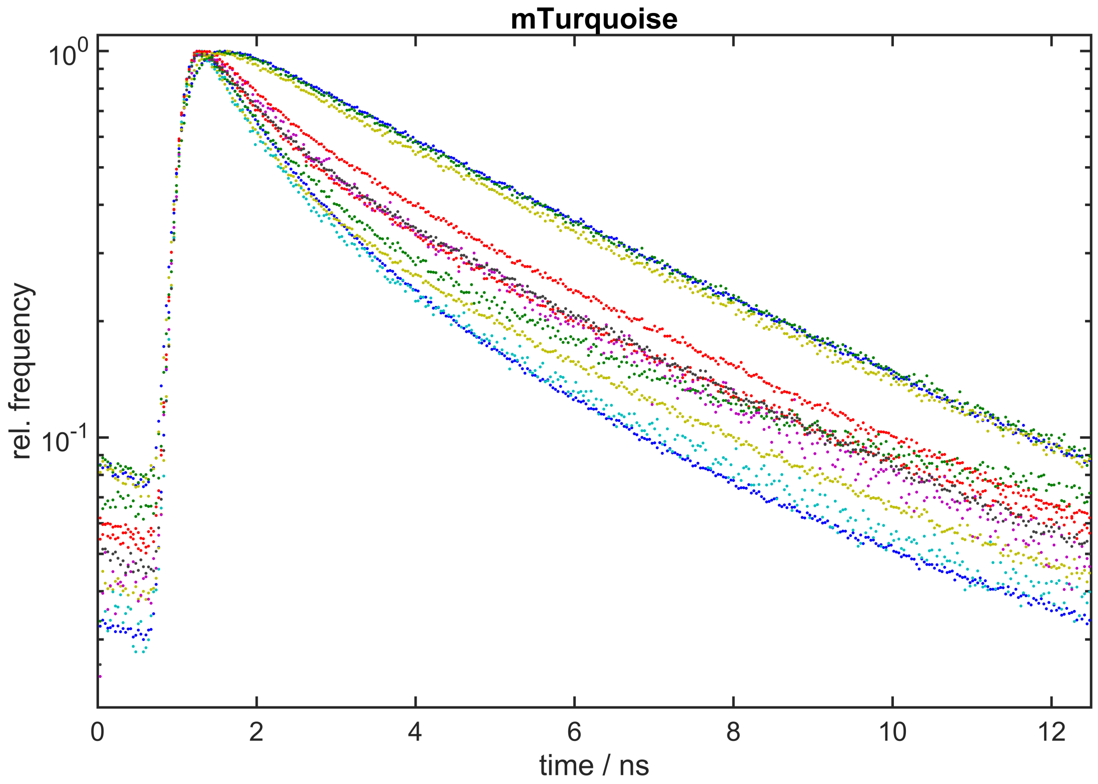


Figure S 13: Fluorescence decays of lifeact-mTurquoise in hMSC cells. Most cells show a mono-exponential decay with a decay-time of about 4.2 ns. Many other cells show bi-exponential decays with a faster component of about 0.7 ns and a slower component of about 3.5 ns. The respective decays show only small variations from cell to cell.

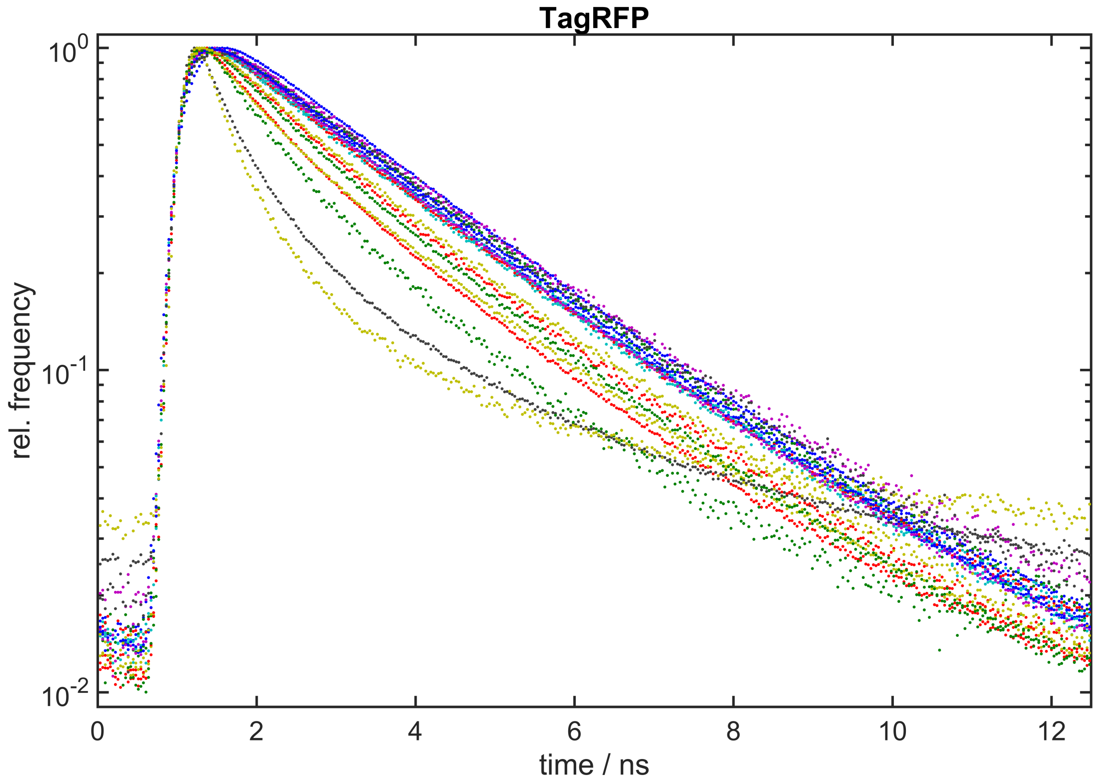


Figure S 14: Fluorescence decays of lifeact-TagRFP in hMSC cells. Some cells show a mono-exponential decay with a decay-time of about 2.4 ns. Many other cells show bi-exponential decays with a faster component varying between 1.5 ns and 0.7 ns.

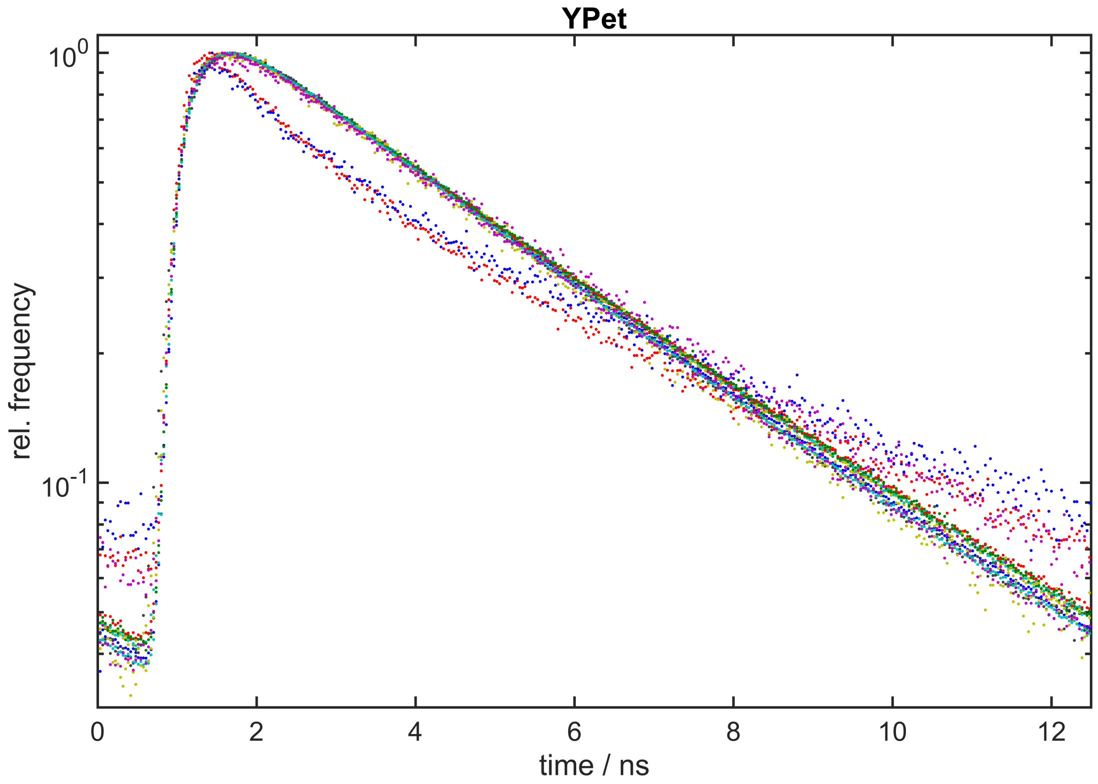


Figure S15: Fluorescence decays of lifeact-YPet in hMSC cells. Most cells show a mono-exponential decay with a decay-time of about 3.5 ns. Some cells show bi-exponential decays with a faster component of about 0.8 ns and a slightly prolonged slower component of about 3.8 ns.

### Present addresses

L.H.: Institute of Pharmacology and Toxicology, University Medical Center Göttingen, Robert-Koch-Str. 40, 37075 Göttingen, Germany

A.G.: Department of Biotechnology and Biophysics, Biocenter, University of Würzburg, Am Hubland, 97074 Würzburg, Germany.

N.K.: Kennedy Institute of Rheumatology, University of Oxford, Oxford OX3 7LF, UK

F.R.: Experimental Physics I, University of Bayreuth, Universitätsstrasse 30, 95440 Bayreuth, Germany.

## References

(1) Hess, S. T.; Sheets, E. D.; Wagenknecht-Wiesner, A.; Heikal, A. A. Quantitative analysis of the fluorescence properties of intrinsically fluorescent proteins in living cells. *Biophysical Journal* **2003**, *85*, 2566–2580.

(2) Patterson, G. H.; Knobel, S. M.; Sharif, W. D.; Kain, S. R.; Piston, D. W. Use of the green fluorescent protein and its mutants in quantitative fluorescence microscopy. *Biophysical Journal* **1997**, *73*, 2782–2790.

(3) Ruhlandt, D.; Andresen, M.; Jensen, N.; Gregor, I.; Jakobs, S.; Enderlein, J.; Chizhik, A. I. Absolute quantum yield measurements of fluorescent proteins using a plasmonic nanocavity. *Communications Biology* **2020**, *3*, 627.

(4) Bindels, D. S.; Haarbosch, L.; van Weeren, L.; Postma, M.; Wiese, K. E.; Mastop, M.; Aumonier, S.; Gotthard, G.; Royant, A.; Hink, M. A.; Gadella, T. W. J. mScarlet: a bright monomeric red fluorescent protein for cellular imaging. *Nature Methods* **2017**, *14*, 53–56.
